# Analysis of the relationship between body habitus and frailty of community adults in Chongqing: a cross-sectional survey study

**DOI:** 10.3389/fpubh.2023.1189173

**Published:** 2023-09-08

**Authors:** Aini Chen, Li Ren, Su Min, Ping Li, Ke Wei, Jun Cao, Yi Tao, Feng Lv

**Affiliations:** ^1^School of Medical, Chongqing Medical University, Chongqing, China; ^2^Department of Anesthesiology, The First Affiliated Hospital of Chongqing Medical University, Chongqing, China; ^3^Department of Phase I Clinical Trial Ward, The First Affiliated Hospital of Chongqing Medical University, Chongqing, China

**Keywords:** frailty, body mass index, waist-hip ratio, body habitus, community adult

## Abstract

**Objective:**

Currently, a multitude of studies are underway to investigate the factors affecting the degree of frailty, with a significant focus on the critical role of body mass index (BMI). This study aims to conduct a cross-sectional survey to investigate the multifaceted relationship between multiple body habitus and the factors that influence the degree of frailty.

**Methods:**

A questionnaire survey was conducted among 840 adult residents in Chongqing communities. A total of 723 participants were included in the data analysis, with an effective response rate of 92.0%. Fried’s frailty scale was used to classify individuals into fit, pre-frail, or frail. Non-parametric tests and chi-square tests were employed to evaluate the inter-group differences in frailty levels under different influencing factors. Multivariate logistic regression analysis was performed to select the independent variables associated with frailty statistics. According to the results of the parallel line test, ordered or disordered multivariate logistic regression was used to evaluate the impact of a single independent variable on frailty for different variables.

**Results:**

Adult community residents in Chongqing accounted for 29.18 and 5.67% in pre-frailty and frailty, respectively. In multivariate logistic regression analysis, high BMI, and high waist-hip ratio (WHR) were identified as major risk factors for frailty. Furthermore, the process of aging, coupled with moderate to heavy alcohol consumption, active weight loss behavior in the past year, and the presence of comorbidities, emerged as significant contributors to frailty. Conversely, factors such as a positive inclination toward taste, consistent meal timing, habitual breakfast consumption, sound nutritional intake, and the cultivation of healthy dietary practices were recognized as pivotal elements that act as protective factors against frailty.

**Conclusion:**

The integration of both BMI and WHR provides a more comprehensive perspective, effectively capturing the intertwined influence of obesity and sarcopenia on the extent of frailty. To mitigate the risk of community-wide frailty, a multipronged approach is essential, involving the promotion of favorable dietary practices and achieving nutritional equilibrium, diligent management of coexisting medical conditions, moderation in alcohol consumption, and the enhancement of physical functionality.

## 1. Introduction

Frailty is a multifaceted clinical condition that arises with age, characterized by a decline in physiological capabilities across various organ systems, rendering individuals more susceptible to stressors (1). The significance of frailty as a predictor of mortality risk among older adults dwelling in the community has been underscored through an in-depth research (2). A comprehensive meta-analysis encompassing 21 studies conducted in high-income countries revealed that the prevalence of frailty among community-dwelling older adults ranged from 4.0 to 59.1%, with an average prevalence of 10.7% (3). In China, the total prevalence rates of pre-frailty and frailty in community-dwelling older adults are 43 and 10%, respectively (4). Clinical manifestations of frailty include weight loss, muscle atrophy and weakness, diminished endurance, compromised balance and mobility, reduced gait speed, and cognitive impairment (5). Given these considerations, it is imperative to embark on research and interventions targeted at addressing frailty.

Beyond the realm of older adults, certain studies have unveiled the occurrence of frailty across other age groups as well (6, 7). Factors affecting frailty are generally considered to be related to BMI (8), as well as risk factors such as aging, smoking, alcohol consumption, comorbidities, malnutrition, diabetes, cognitive impairment, and history of falls (9). Frailty can be mitigated through engagement in physical activities, exercise regimens, dietary modifications, and modulation of gut microbiota (10–13).

Previous research (14) has shown that sarcopenia (the decline in function accompanied by low muscle mass) is the primary cause of frailty, accounting for 70% of frailty cases. The Asian Working Group for Sarcopenia (AWGS) 2014 consensus has defined sarcopenia as “age-related loss of muscle mass, plus low muscle strength, and/or low physical performance” (15), the European Society for Clinical Nutrition and Metabolism (ESPEN) in association with the European Association for the Study of Obesity (EASO) considers BMI as a screening tool for sarcopenia, highlighting that excessive adiposity in conjunction with diminished skeletal muscle mass or correlated body compartments substantiates the diagnosis of sarcopenia (16). Since sarcopenia is a prominent manifestation and risk factor for frailty, BMI is frequently adopted in studies to prognosticate the extent of frailty. Low BMI has been correlated with an elevated frailty risk (17), while conversely, an elevated BMI might augment the likelihood of frailty (18). Nevertheless, relying solely on BMI might not yield a comprehensive understanding of the degree of frailty. Hence, the incorporation of additional indicators of body habitus, such as waist-hip ratio, body fat percentage, body roundness index, body shape index, etc. Become crucial for defining individual physical characteristics, including physique, general bearing, and body shape (19). These indicators facilitate a more accurate and comprehensive characterization of frail patients. Furthermore, scant exploration has been devoted to the factors that influence body habitus, such as dietary habits and nutritional status. Therefore, this study aimed to explore the relationship between body habitus (BMI and the degree of frailty), potential associations between lifestyle, nutrition, dietary habits, and the degree of frailty [as indicated by waist-hip ratio (WHR), body fat percentage (BFP), body roundness index (BRI), and body shape index (ABSI)] based on a community sample.

## 2. Materials and methods

### 2.1. Ethical considerations

This study followed the guidelines for human research in the 2013 revised Declaration of Helsinki. The research protocol was approved by the Ethics Committee of the First Affiliated Hospital of Chongqing Medical University (2023–021) and registered at the Chinese Clinical Trial Registry in 2023 (ChiCTR2300068834). Participation in the survey was entirely voluntary, and prior to the commencement of the survey, written informed consent was obtained from each participant. It was explicitly communicated that the anonymity of all participants would be rigorously upheld.

### 2.2. Participants and procedure

The present cross-sectional study was carried out in Chongqing (China) from March 1, 2023 to March 7, 2023. To be eligible for participation, individuals had to meet the following inclusion criteria: (1) Possession of a registered household registration or temporary residency permit in Chongqing, with a minimum residency duration of 6 months, excluding those classified as migrant population; (2) Attainment of at least 18 years of age; and (3) Willingness to provide informed consent for participation in the study. In order to ensure the scientific validity of the survey design, a multi-stage random cluster sampling method was employed, taking into account significant aspects such as economic status and demographic variables. This approach aimed to ensure the overall representativeness of the sample by aligning with regional income standards, maintaining a distribution of age and gender akin to the general population in Chongqing, and achieving a balanced geographical distribution. The survey targeted 10 communities within Chongqing, which were selected through a random sampling process. Among 12 street offices, six were chosen at random, and from each selected street office, three communities were randomly picked. The sample size of each community survey was approximately 40 individuals. Ultimately, three street offices were identified for inclusion in the survey. All individuals meeting the inclusion criteria were selected from the designated households until the requisite sample size was attained. The study population encompassed individuals aged 18 or above, inclusive of those aged 18. A total of 720 participants were required in the 10 surveyed communities. Upon obtaining informed consent from the participants, pertinent data was acquired *via* on-site survey questionnaires, which were pre-tested to ensure their validity and reliability. In total, 840 survey questionnaires were distributed, yielding 786 collected responses and an actual response rate of 93.6%. After the exclusion of 61 participants with missing data, the final dataset comprised 723 participants (*n* = 723), resulting in an effective rate of 92.0%.

### 2.3. Measures

The survey encompassed an assessment of various domains, including socio-demographic information, body habitus, nutritional assessment, dietary habits, Fried’s frailty scale, and related domains (Appendix 1). Trained personnel executed all evaluations using standardized methodologies. Before distributing the questionnaire, a panel of 5 experts participated in the discussion and evaluation process for scrutinizing and refining the language, accuracy, order, and fluency of the questions and response options. The evaluation process comprised both individual assessment and group discussion. To obviate mutual influence, the experts remained uninformed about each other’s evaluation outcomes. Following the expert evaluation, the questionnaire underwent an initial pre-testing within a small-scale community study, yielding a Cronbach’s alpha coefficient of 0.744.

#### 2.3.1. Sociodemographic characteristics

This study thoroughly examined the sociodemographic characteristics of the participants, including age, gender, marital status (single, married, divorced, widowed), education level (primary school and below, junior high school, senior high school, vocational school, undergraduate, graduate and above), occupation (government enterprises, healthcare professionals, self-employed, students, and retired) and personal income (less than ¥1,000, ¥1,000–3,999, ¥4,000–9,999, more than ¥10,000). Smoking status was classified as smoking (continuous or cumulative smoking for 6 months or more) or non-smoking (20). Drinking status was classified as non-drinking, moderate drinking (female ≤1 glass/day for women, male ≤2 glasses/day), or heavy drinking (female >1 glass/day, male >2 glasses/day) (21). Exercise was defined as moderate-intensity activity for ≥150 min/week or high-intensity activity for ≥75 min/week (22). Active weight loss behavior in the past year was defined as a binary variable (yes or no), indicating whether individuals had actively pursued strategies to manage their weight. Comorbidities were defined as self-reported chronic diseases (such as hypertension, diabetes, coronary heart disease, cerebrovascular disease, chronic obstructive pulmonary disease, joint diseases, etc.).

#### 2.3.2. Body habitus

This section comprises nine indicators pertinent to body habitus, including height, weight, waist circumference, hip circumference, BMI, WHR, BFP, BRI, and ABSI.

BRI serves as an index for measuring the distribution of body fat, which considers the mass, distribution, and shape of total fat. BRI is calculated by the ratio of waist circumference to hip circumference. A higher BRI value corresponds to greater fat accumulation around the waist. Notably, BRI stands as a more precise gage of waist fat distribution and the roundness of body shape, characterized by robust practicality and accuracy (23, 24). ABSI, on the other hand, offers a body shape measurement that takes into account body fat distribution and BMI (25). ABSI is calculated based on the ratio of waist circumference to [height raises to 2/3 power times the square root of BMI]. A higher ABSI value implies greater waist fat accumulation relative to height and BMI. Several studies have shown that high ABSI and BRI values are associated with increased risks of cardiovascular disease, diabetes, and cancer (26, 27). Currently, ABSI is incorporated in certain studies for the prediction of health risks and the evaluation of body shape (28). The measurement of all the aforementioned indicators adhered to the standard methods specified by the Chinese Center for Disease Control and Prevention (29). Calibrated weighing scales with a precision of 1,000 grams were employed, height measurements were precise to 1 cm, and the calculation outcomes were recorded to two decimal places.

#### 2.3.3. Nutritional assessment scale

The Short Nutritional Assessment Questionnaire (SNAQ), a concise, succinct, and repeatable survey questionnaire, was utilized for the nutritional appetite assessment (30). SNAQ consists of 4 questions, each with 5 answer options, represented by the letters A through E. The questionnaire’s content, along with the scoring criteria, are detailed in Appendix 1. Lower scores denote poorer appetite and an elevated risk of weight loss, with the score range spanning 4 to 20. A score of ≤14 signifies a potential risk of weight loss.

#### 2.3.4. Dietary habits assessment scale

This segment of the questionnaire aligns with the “Chinese Dietary Guidelines for Residents (2016 edition)” (31). The questionnaire consists of 5 questions, and the specific questions and corresponding scoring criteria can be found in Appendix 1. The cumulative score on this scale ranges from 12 to 55 points.

#### 2.3.5. Frailty assessment scale

In this study, frailty measurement was defined using the Fried and colleagues Frailty Assessment Scale (32), a rigorously validated and widely used tool. The assessment model of this scale centers around five criteria: feeling exhausted, physical activity, walk time, grip strength, and weight loss. In instances where a participant is unable to ambulate due to a fracture or injury, their pre-fracture or pre-injury activity level should be reported. Satisfying 1–2 of these criteria indicates pre-frailty while meeting 3 or more criteria signifies frailty.

### 2.4. Statistics analysis

IBM SPSS Statistics 26.0 (IBM Corporation, Armonk, NY, United States) was used for data analysis. First, descriptive statistics were used to examine the baseline characteristics of the study population. Next, the Kruskal-Wallis H test was used for non-parametric analysis of asymmetric continuous count data, while the chi-square test was used for metric data of continuous normal distribution. These assessments were conducted to discern whether variations existed in the distribution of different frailty degrees across diverse conditions, including body habitus, nutritional status, and dietary habits. Then, multiple logistic regression was performed to identify the most significant frailty-related predictors among variables exhibiting statistical significance. Finally, based on parallel line test results, ordinal or nominal multiple logistic regression analyzes were conducted for each variable with statistical differences to examine the relationship between the specific predictor variable and frailty. In the regression analyzes, all variables considered to be potential confounders were meticulously adjusted. All statistical tests conducted within the model were two-tailed and predicated on a significance level of 0.05.

## 3. Result

### 3.1. Participants’ sociodemographic characteristics

Table 1 presents the socio-demographic characteristics of the 723 participants. The median age of the sample was 47 years, with a first quartile (Q1) of 29 and a third quartile (Q3) of 67 (SD = 20.217, SE = 0.752). Among the participants, 51.5% were females, while 48.5% were males. Out of the total, 471 individuals (65.1%) were considered healthy, 211 (29.2%) exhibited pre-frailty, and 41 (5.7%) were identified as frail. Regarding occupational distribution, a majority of participants were engaged in company jobs (31.3%) or had retired (34.3%). As for education, 39.6% possessed a college degree. In terms of monthly income, the largest proportion fell within the range of 1,001–3,499 yuan (31.1%). Additionally, 40.1% of the participants were married.

**Table 1:** Baseline characteristics adjusted for debilitating status (n = 723).

Characteristics		Fit (*n* = 471)		Pre-frail (*n* = 211)		Frail (*n* = 41)		Total (*n* = 723)	
		*N*	%	*N*	%	N	%	*N*	%
Gender	Female	260	55.2%	94	44.5%	18	43.9%	372	51.5%
	Male	211	44.8%	117	55.5%	23	56.1%	351	48.5%
Age*	Below 29	139	29.5%	19	9.0%	1	2.4%	159	22.0%
	29 ~ 47	140	29.7%	52	24.6%	8	19.5%	200	27.7%
	47 ~ 67	100	21.2%	67	31.8%	11	26.8%	178	24.6%
	Above 67	92	19.5%	73	34.6%	21	51.2%	186	25.7%
Profession	Self-employed	111	23.6%	50	23.7%	5	12.2%	166	23.0%
	Company Enterprise	162	34.4%	55	25.1%	9	21.9%	226	31.3%
	Health Care	39	8.3%	1	0.5%	0	0.0%	40	5.5%
	Student	39	8.3%	4	1.9%	0	0.0%	43	5.9%
	Retiree	120	25.5%	101	47.9%	27	65.9%	248	34.3%
Educational background	Primary school and below	1	0.2%	3	1.4%	2	4.9%	6	0.8%
	Junior high school	95	20.2%	65	30.8%	14	34.1%	174	24.1%
	High School	133	28.2%	57	27.0%	10	24.4%	200	27.7%
	Undergraduate	198	42.0%	75	35.5%	13	31.7%	286	39.6%
	Postgraduate and above	44	9.3%	11	5.2%	2	4.9%	57	7.9%
Monthly incomes (RMB, Yuan)	≤1,000	70	14.9%	36	17.1%	6	14.6%	112	15.5%
	1,001–3,499	52	32.5%	37	26.1%	7	41.5%	96	31.1%
	3,500–9,999	153	11.0%	55	17.5%	17	17.1%	225	13.3%
	≥10,000	117	24.8%	53	25.1%	4	9.8%	174	24.1%
Marital status	Single	194	41.2%	59	28.0%	10	24.4%	263	36.4%
	Married	176	37.4%	96	45.5%	18	43.9%	290	40.1%
	Divorced	81	17.2%	42	19.9%	9	22.0%	132	18.3%
	Widowed	20	4.2%	14	6.6%	4	9.8%	38	5.3%

*Segmentation of age according to the results of the quartiles.

### 3.2. Comparison of body habitus and influencing factors for frailty

Table 2 demonstrates the comparison and tests conducted between body habitus and levels of frailty. After standardized grouping based on diverse indicators, the populations categorized as frail and pre-frail were primarily concentrated within the overweight population (75.6%). Individuals exhibiting both high and low WHR manifested varying degrees of frailty, with a higher risk of frailty observed in comparison to those with normal WHR. Notably, among individuals with different levels of frailty, BMI, WHR, BRI, and ABSI demonstrated significant inter-group differences (*p* < 0.05), suggesting their potential influence on frailty degree. Moreover, the results indicated significant group variations within the Nutrition Assessment Scale and the Dietary Habits Assessment Scale, as reflected by distinct total scores (p < 0.05) among individuals with varying degrees of frailty. Specifically, factors such as the acceptance of food taste, regular daily meals, and regular breakfast may become one of the factors affecting the degree of frailty. Additionally, diverse lifestyles were found to have an impact on the degree of frailty. Variables such as smoking, alcohol consumption, active weight loss behavior in the past year, patient age, and the presence of comorbidities were all associated with varying degrees of frailty within different populations.

**Table 2:** Descriptive analysis of body habitus and influencing factors.

Independent variable		Fit (*n* = 471)	Pre-Frail (*n* = 211)	Frail (*n* = 41)	Total (*n* = 723)	*P*
		*N*(%)/Mean (SD)	*N*(%)/Mean (SD)	*N*(%)/Mean (SD)	*N*(%)/Mean (SD)	
Body habitus						
Body mass index (BMI)	Underweight	29 (6.20%)	4 (1.90%)	0 (0.00%)	33 (4.60%)	0.000*
	Normal weight	217 (46.10%)	64 (30.30%)	10 (24.40%)	291 (40.20%)	
	Overweight	225 (47.80%)	143 (67.80%)	31 (75.60%)	399 (55.20%)	
Waist-hip ratio (WHR)	Low	127 (27.00%)	67 (31.80%)	7 (17.10%)	201 (27.80%)	0.010*
	Normal	97 (20.60%)	38 (18.00%)	2 (4.90%)	137 (18.90%)	
	High	247 (52.40%)	106 (50.20%)	32 (78.00%)	385 (53.30%)	
Body fat percentage	Low	1 (0.20%)	3 (1.40%)	0 (0.00%)	4 (0.60%)	0.083*
	Normal	30 (6.40%)	2 (0.90%)	2 (4.90%)	34 (4.70%)	
	High	440 (93.40%)	206 (97.60%)	39 (95.10%)	685 (94.70%)	
Body roundness index (BRI)		3.26 ± 0.62	2.99 ± 0.76	3.08 ± 0.61	3.17 ± 0.68	0.000
A body shape index (ABSI)		0.73 ± 0.11	0.69 ± 0.14	0.72 ± 0.11	0.72 ± 0.12	0.038
Lifestyle						
Age		44.17 ± 19.60	56.22 ± 18.78	63.61 ± 15.60	48.79 ± 20.22	0.000
Smoking^**^	No	321 (68.20%)	95 (45.00%)	20 (48.80%)	436(60.30%)	0.000
	Yes	150 (31.80%)	116 (55.00%)	21 (51.20%)	287 (39.70%)	
Drinking alcohol^***^	No	369 (78.30%)	127 (60.20%)	22 (53.70%)	518 (71.60%)	0.000
	Moderate drinking	69 (14.60%)	53 (25.10%)	15 (36.60%)	137 (18.90%)	
	Heavy drinking	33 (7.00%)	31 (14.70%)	4 (9.80%)	68 (9.40%)	
Active weight loss behavior in the past year^****^	No	416 (88.30%)	177 (83.90%)	31 (75.60%)	624 (86.30%)	0.036
	Yes	55 (11.70%)	34 (16.10%)	10 (24.40%)	99 (13.70%)	
Daily exercise^*****^	No	183 (38.90%)	83 (39.30%)	18 (43.90%)	284 (39.30%)	0.818
	Yes	288 (61.10%)	128 (60.70%)	23 (56.10%)	439 (60.70%)	
Comorbidities	No	413 (87.70%)	61 (28.90%)	16 (39.00%)	490 (67.80%)	0.000
	Yes	58 (12.30%)	150 (71.10%)	25 (61.00%)	233 (32.20%)	
SNAQ total score		13.42 ± 2.66	12.88 ± 2.87	13.17 ± 2.70	13.35 ± 2.73	0.020
Appetite level		3.44 ± 1.23	3.29 ± 1.32	3.56 ± 1.25	3.4 ± 1.25	0.338
Food taste acceptance		3.4 ± 1.22	3.12 ± 1.23	3.05 ± 1.32	3.3 ± 1.23	0.009
Food intake per meal		3.13 ± 1.15	3.03 ± 1.2	3.1 ± 1.02	3.1 ± 1.16	0.497
Number of meals per day		3.45 ± 0.98	3.44 ± 1.1	3.46 ± 1.03	3.45 ± 1.02	0.971
Dietary habits scale total score		39.12 ± 5.04	38.24 ± 6.96	37.78 ± 5.261	38.79 ± 5.691	0.002
Daily meal situation		1.63 ± 0.48	1.5 ± 0.5	1.39 ± 0.49	1.58 ± 0.49	0.000
Regular breakfast		2.59 ± 0.51	2.4 ± 0.56	2.44 ± 0.55	2.53 ± 0.53	0.000
Eating frequency of staple food		4.13 ± 1.01	3.92 ± 1.23	4.1 ± 0.94	4.07 ± 1.07	0.254
Eating frequency of whole grains		3.1 ± 1.12	3.23 ± 1.35	3.32 ± 1.04	3.15 ± 1.19	0.285
Eating frequency of vegetables		3.71 ± 1.15	3.68 ± 1.22	3.27 ± 1.27	3.68 ± 1.18	0.084
Eating frequency of fruit		3.59 ± 1.19	3.62 ± 1.27	3.54 ± 1.23	3.6 ± 1.21	0.821
Eating frequency of poultry		3.5 ± 1.18	3.53 ± 1.24	3.61 ± 1.12	3.51 ± 1.19	0.855
Eating frequency of meat		3.11 ± 1.28	2.95 ± 1.31	2.85 ± 1.17	3.05 ± 1.29	0.199
Eating frequency of fish and its products		3.27 ± 1.3	3.34 ± 1.31	3.12 ± 1.29	3.28 ± 1.3	0.570
Eating frequency of eggs and its products		3.65 ± 1.13	3.57 ± 1.21	3.34 ± 1.32	3.61 ± 1.17	0.406
Eating frequency of beans and its products		3.44 ± 1.26	3.18 ± 1.32	3.39 ± 1.12	3.36 ± 1.27	0.054
Eating frequency of milk and its products		3.39 ± 1.25	3.33 ± 1.37	3.41 ± 1.22	3.37 ± 1.28	0.946

*Since there is a certain categorical sample size n < 5 for this categorical variable, Fisher’s exact test is used. **Smoking: Smoking is defined as continuous or cumulative smoking for 6 months or more. ***Drinking alcohol: Whether it is white wine, beer, wine, or yellow wine, as long as the number of times it is consumed is defined as drinking alcohol, and those who consume it only once on festivals are not considered as drinking alcohol. ****Active weight loss behavior in the past year: It is defined as a binary variable (yes or no), indicating whether individuals had actively pursued strategies to manage their weight. It may not always result in significant weight reduction and can be influenced by various factors. These factors could include medical conditions, medications, and other underlying health conditions. *****Daily exercise: physical activity was defined as ≥150 min/week of moderate-intensity or ≥ 75 min/week of high-intensity physical activity.

### 3.3. Multivariate logistic regression analysis of factors related to different degrees of frailty

Using frailty, pre-frailty, and fitness as dependent variables, we considered the following factors as independent variables: age, smoking, drinking, active weight loss behavior in the past year, comorbidities, BMI, WHR, BRI, ABSI, food taste acceptance score, daily regular meal score, regular breakfast score, total score of Nutrition Assessment Scale and total score of Dietary Habits Scale. Table 3 shows the variables that exhibited statistical significance in the analysis (*p* < 0.05). In our study, the application of multivariate logistic regression aims to identify significant contributing factors without establishing linear relationships between variables. This statistical approach is commonly employed in the field (33, 34). owing to its effectiveness in identifying significant predictors among multiple variables. It serves as a valuable tool for assessing associations between various factors and the targeted outcome. The results showed that 11 independent variables were associated with frailty and pre-frailty, including age, moderate and heavy drinking, active weight loss behavior in the last year, comorbidities, BMI, WHR, food taste acceptance score, daily meal score, regular breakfast score, total score of the Short Nutritional Assessment Scale, and total score of the Eating Habits Scale.

**Table 3:** Multivariate logistic regression analysis of factors related to different degrees of frailty.

Independent variable		Pre-Frail vs. Fit				Frail vs. Fit			
		OR	95%CI		*P*	OR	95%CI		*P*
			Lower	Upper			Lower	Upper	
Body habitus									
Body mass index (BMI)	Normal weight(ref)								
	Underweight	0.621	0.142	2.712	0.526	2.829E-09	2.829E-09	2.829E-09	0.000
	Overweight	0.615	1.191	0.603	2.351	3.527	1.013	12.286	0.048
Waist-hip ratio (WHR)	Normal(ref)								
	Low	1.073	0.557	2.065	0.833	2.228	0.407	12.189	0.355
	High	1.423	0.783	2.585	0.247	10.265	2.165	48.667	0.003
Body roundness index (BRI)		0.967	0.139	6.748	0.973	5.615	0.206	153.403	0.307
A body shape index (ABSI)		0.030	1.640E-06	554.665	0.485	3.965E-05	1.764E-12	891.252	0.241
Lifestyle									
Age		1.005	0.991	1.019	0.490	1.048	1.024	1.072	0.000
Smoking	No(ref)								
	Yes	0.666	0.419	1.060	0.087	0.689	0.311	1.525	0.358
Drinking alcohol	No(ref)								
	Moderate drinking	1768	1.019	3.066	0.043	3.105	1.336	7.217	0.008
	Heavy drinking	2.189	1.089	4.402	0.028	1.389	0.393	4.911	0.610
Active weight loss behavior in the past year	No(ref)								
	Yes	1.850	1.008	3.394	0.047	2.512	0.971	6.501	0.058
Comorbidity	No(ref)								
	Yes	23.528	13.649	40.556	0.000	8.147	3.347	19.831	0.000
SNAQ total score		1.079	0.968	1.202	0.168	1.240	1.029	1.495	0.024
Food taste acceptance score		0.837	0.671	1.043	0.113	0.692	0.481	0.996	0.047
Dietary habits scale total score		1.063	1.018	1.111	0.006	1.058	0.985	1.138	0.124
Daily meal score		0.614	0.393	0.959	0.032	0.360	0.164	0.788	0.011
Regular breakfast score		0.355	0.234	0.539	0.000	0.553	0.270	1.133	0.106

### 3.4. Logistic regression analysis of the single factor and different degrees of frailty

To prevent mutual influence and interference among independent variables, each independent variable was subjected to separate regression analysis. Previously, a parallel lines assumption test was conducted to ensure the parallelism of the response curves of categorical variables in the ordered logistic regression model. This parallel lines assumption, commonly used in cross-sectional studies (35), signifies that distinct categories of an independent variable maintain a consistent parallel relationship across its entire range. This ensures the reliability of regression models for prediction and interpretation. If the parallel lines assumption is violated (*p* < 0.05), it suggests that the model may not accurately capture the relationships between different categories. This situation can lead to unstable predictive outcomes and compromise the overall reliability of the model. When *p* > 0.05, the response curves of each variable at varying levels of categorical variables exhibit parallelism, which can be analyzed using an ordered logistic regression model (36, 37).

As a result, except for WHR and comorbidities analyzed using unordered logistic regression, all other independent variables can be analyzed using ordered logistic regression. The impact of each independent variable on the degree of frailty was statistically different (*p* < 0.05). The results presented in Tables 4, 5 showed that aging annually increases the log odds value of frailty by 0.035 units (B = 0.035), indicating the significant effect of age on frailty. In terms of body habitus, the degree of frailty increased with increasing BMI values. The coefficient B for the variable “Overweight” with a value of 0.823 (greater than 0) indicates that a positive value signifies an augmented probability ratio of transitioning from lower to higher levels of the dependent variable with an increase in the independent variable. This implies that the degree of frailty is correlated with an elevated BMI. For every unit increase in the BMI value, the odds ratio of transitioning from one level of the dependent variable to the next higher level increases by approximately a factor of 0.823. Individuals with a high WHR had a 6.283-fold increased risk of frailty compared to fitness. Moderate drinking, heavy drinking, and comorbidities, as factors influencing body habitus, all increased the relative probability of frailty (B > 0). Conversely, the relative probability of frailty decreased (B < 0) with each unit increase in active weight loss behavior in the past year, food taste acceptance score, daily meal score, regular breakfast score, simple nutritional assessment scale total score, and dietary habit scale total score. Comorbidity significantly increased the odds of frailty (OR = 11.126) and pre-frailty (OR = 17.510) when compared to the healthy population, and these results were statistically significant (*p* < 0.01).

**Table 4:** Univariate ordinal logistic regression.

Independent variable		*B*	95%CI		*P*
			Lower	Upper	
Age		0.035	0.027	0.043	0.000
Drinking alcohol	No(ref)				
	Moderate drinking	0.918	0.543	1.293	0.000
	Heavy drinking	0.886	0.389	1.383	0.000
Active weight loss behavior in the past year	No(ref)				
	Yes	0.508	0.089	0.927	0.018
Body mass index (BMI)	Normal weight(ref)				
	Underweight	−0.921	−2.004	0.163	0.096
	Overweight	0.832	0.496	1.149	0.000
SNAQ total score		−0.061	−0.116	−0.006	0.031
Food taste acceptance score		−0.188	−0.311	−0.066	0.003
Dietary habits scale total score		−0.029	−0.056	−0.002	0.035
Daily meal score		−0.639	−0.945	−0.333	0.000
Regular breakfast score		−0.599	−0.883	−0.315	0.000

**Table 5:** Univariate multinomial logistic regression.

Independent variable		Pre-Frail vs. Fit				Frail vs. Fit			
		OR	95%CI		*P*	OR	95%CI		*P*
			Lower	Upper			Lower	Upper	
Comorbidities	No(ref)								
	Yes	17.510	11.677	26.256	0.000	11.126	5.608	22.073	0.000
Waist-hip ratio (WHR)	Normal(ref)								
	Low	1.347	0.835	2.171	0.222	2.673	0.543	13.115	0.227
	High	1.095	0.706	1.699	0.684	6.283	1.477	26.726	0.013

## 4. Discussion

In previous studies, discussions on frailty mainly centered on the older adult population (38, 39). The present study conducted a comprehensive analysis of adult community residents for the first time. The outcomes unveiled a frailty prevalence rate of 5.67% and a pre-frailty prevalence rate of 29.18%, which were consistent with findings from numerous previous studies (40, 41). Moreover, this research delved into body habitus indicators beyond the confines of BMI, opting for a more accurate and comprehensive evaluation through multiple indicators such as WHR, body fat percentage, BRI, and weight index. This multifaceted approach enables a more comprehensive grasp of the relationships between factors such as fat mass, central obesity, and frailty.

In this study, Fried’s frailty scale (32), also known as the phenotype model (42), was utilized as the tool for screening and grading frailty. As the most widely employed method for assessing frailty, this questionnaire adeptly and swiftly portrays participants’ physical frailty statuses. Additionally, it exhibits a robust correlation with BMI and other body habitus indicators, rendering it the most appropriate method for assessing frailty within the scope of this cross-sectional study (43). Although the Fried frailty scale was initially designed for individuals aged 65 and older, recent research has indicated that it maintains a high level of accuracy when applied to the assessment of frailty in the middle-aged population (44). In contrast, the commonly used clinical frailty scale (CFS) (45) and frailty index (FI) (46) lack comprehensive evaluation criteria pertaining to body habitus. Consequently, these tools possess certain limitations when evaluating frailty across diverse body habitus profiles. The CFS primarily assesses frailty based on mobility status, while the FI lacks standardized criteria for variable inclusion. Researchers determine variable inclusion based on their study objectives and available health indicators, leading to variations in the number of variables (ranging from 30 to 70), and the corresponding threshold values (47–49), and researchers from different countries have developed their own versions of the FI. Hence, these two commonly used assessment tools inherently carry limitations that render them unsuitable for the present study.

Nutritional status was evaluated using the Simplified Nutritional Appetite Questionnaire (SNAQ) (50), which encompasses several indicators such as appetite, hunger, and sensory perception. This questionnaire embraces high reliability, sensitivity, and predictability, enabling it to effectively reflect the nutritional status of the population and predict the risk of malnutrition. To evaluate dietary habits, the China Food Guide Pagoda (2016 version) (31) and multiple references were consulted, covering various types of food and frequency of consumption, providing a comprehensive reflection of participants’ dietary preferences and dietary balance.

The findings of this study reaffirmed previously identified risk factors for frailty, including age, alcohol consumption, and comorbidities (4, 9, 14). In comparison to individuals classified as fit, those categorized as frail or pre-frail were older and more likely to engage in moderate or heavy drinking and suffer from comorbidities. Prior research has indicated that exercise can mitigate the risk of frailty and enhance physiological function in older adults (51). However, in this study, 56.10% of frail participants and 60.70% of pre-frail participants reported engaging in regular exercise. This suggests that the manner, frequency, and intensity of their exercise may not be sufficient to effectively prevent or delay frailty. Furthermore, these results imply that the cause of frailty may extend beyond merely insufficient exercise, encompassing other factors such as age, health status, dietary habits, and medication usage.

In the present study, the relationship between BMI and frailty did not fully align with the findings of previous research. According to one study (52), there is a U-shaped association between BMI and frailty, suggesting that both high and low BMI values increase the likelihood of frailty. In our study, overweight individuals had a higher incidence of frailty, but the statistical association between low BMI value and frailty was not fully confirmed. Several factors could contribute to this outcome. Firstly, due to the high prevalence of obesity in China (53), our study had limited representation of individuals with low BMI and instead, had a greater prevalence of overnutrition and high BMI. This trend is consistent with previous research conducted within Chinese communities (54). Moreover, this discrepancy might be explicable through the mechanisms of frailty. Sarcopenia, the key contributor to frailty, primarily involves a decline in muscle mass within the body (55). However, for participants with lower BMI values, the reduction in BMI might not be entirely attributed to the loss of muscle mass; it could also involve loss of fat or calcium (56, 57). Similarly, a high BMI does not necessarily signify muscle gain but may be a sign of fat gain and muscle loss. Numerous studies have highlighted the risk of synergistic complications of sarcopenia and obesity in aging populations (58). Therefore, if frailty is described solely from the perspective of BMI as in previous studies, the relationship between sarcopenia and frailty may not be adequately substantiated. Our study encompassed various body habitus indicators, and both BMI and WHR demonstrated statistical significance. As a descriptive index for waist and hip circumference, WHR can better reflect the distribution of muscle and fat in different parts of the body. It effectively highlights unfavorable fat accumulation around the abdomen and is also easier to calculate than BMI (59). Several studies have demonstrated that the WHR is a more effective predictor of mortality and the incidence of certain diseases in middle-aged and older adults compared to BMI. It has also been adopted as an indicator of health and the presence of significant health risks (60, 61). Our results confirmed that high WHR independently increased the risk of current community frailty. This finding suggests that, in the context of Chinese community populations, low BMI or low WHR no longer constitute major factors in frailty development. Instead, high BMI and high WHR are more closely associated with frailty progression. Furthermore, we propose that WHR can offer a more accurate depiction of the relationship between frailty and sarcopenia compared to BMI. Individuals with high WHR are more likely to shift from fitness to frailty, implying an overlap between our physical condition characterized by high waist fat and low hip circumference fat, and the clinical manifestations of sarcopenia. Unlike BMI, which solely considers height and weight, WHR takes into account critical factors of sarcopenia: muscle mass and fat mass. It is worth noting that the formula for calculating WHR considers gender differences, which is particularly important since females are more susceptible to frailty (3, 62, 63). This gender-specific aspect is often overlooked when calculating BMI. In conclusion, the assessment of frailty can be enhanced by considering multiple body habitus indicators, such as WHR and BMI, to achieve more accurate and comprehensive results.

The absence of a significant correlation between body fat percentage (BFP) and frailty status, as observed in Table 2, presents an intriguing finding. This finding aligns with previous research that has highlighted inconsistencies and even reversals in the significance of WHR and BFP (64). BFP serves as an indicator of the proportion of total body weight attributed to adipose tissue, essentially representing the overall adiposity of the body. While an elevated body fat percentage may indicate a higher overall fat content, it fails to provide insights into the distribution of fat within specific anatomical regions. In the context of our study, the lack of a significant association between body fat percentage and frailty status suggests that the overall fat content might have a relatively minor impact on frailty. Conversely, WHR is regarded as a more refined measure, reflecting the proportion of abdominal fat within the total body fat composition. Elevated WHR is often indicative of abdominal obesity, which has a strong association with various health concerns and displays a significant relationship with frailty status (65). Our study confirms this perspective, underscoring that WHR could be more sensitive than BFP in assessing frailty status, or alternatively, the abdominal fat distribution might play a more pivotal role in determining frailty status. Furthermore, studies have indicated that Asians tend to possess a higher proportion of visceral fat compared to Europeans, which provides an additional perspective to explain the significant WHR result and the non-significant BFP finding in our study (66, 67).

This study also covered some relevant factors affecting body habitus, including lifestyle, nutritional status, and dietary habits. The scoring outcomes of the questionnaire imply that a regimen of regular eating, daily breakfast consumption, high acceptance of food taste, a balanced diet, and adequate nutrition can collectively lower the relative likelihood of developing frailty. This suggests that favorable dietary habits can contribute to weight management, maintaining health status, and diminishing the risk of frailty (68, 69). Additionally, some literature has suggested a relationship between frailty and malnutrition (70–72). Malnutrition has been identified as the primary risk factor for frailty among community-dwelling older adults (73), which has a greater impact on the frailty of the population (70).

## 5. Limitation

Although we selected the most suitable frailty assessment scale for our study, there is currently no frailty scale available that encompasses all age groups. Hence, there might be some bias in the assessment of frailty among the younger population. In terms of sample selection and collection, this study was limited to community populations in urban areas of China. While we have made considerable efforts to ensure sample representativeness, achieving a 100% representation of the overall situation is challenging. Moreover, factors such as rural areas and ethnicity should be taken into consideration. Additionally, the sample size gathered for some indicators remains moderate. Despite our efforts to rule out collinearity, this might result in some inevitable errors in the outcomes, such as wider confidence intervals. In future investigations, we intend to explore the possibility of employing a multi-center or longitudinal research approach, thereby broadening the spectrum and comprehensiveness of the sample. Furthermore, the percentage of body fat was calculated during the measurement process, without using instruments for obtaining highly accurate results. To enhance the accuracy of body composition measurement, we aim to employ more comprehensive and precise measurement methods. Finally, since our study was cross-sectional in nature, we did not conduct subgroup analyzes of frailty status across different populations or genders. Further research is needed to explore the interplay and influence of BMI and other contributing factors.

## 6. Conclusion

This cross-sectional analysis elucidated the relationship between body habitus and frailty in community-dwelling adults. The study also incorporated pertinent influencing factors such as lifestyle, nutritional status, and dietary habits. The results demonstrated that compared to previous studies solely focused on BMI, a comprehensive evaluation of body habitus involving BMI, WHR, BFP, BRI, ABSI, and other indicators can provide a more thorough and accurate reflection of the influence of body size and obesity on frailty severity. Furthermore, this approach can help explain the mechanisms underlying frailty caused by sarcopenia, thereby aiding in predicting changes in the prevalence of adult frailty within communities. By fostering good dietary habits, achieving nutritional balance, managing comorbidities, and reducing alcohol consumption, the risk of community frailty can be significantly mitigated. These findings offer novel insights into targeted prevention and even reversal of frailty.

## Data availability statement

The raw data supporting the conclusions of this article will be made available by the authors, without undue reservation.

## Ethics statement

The studies involving humans were approved by the Ethics Committee of the First Affiliated Hospital of Chongqing Medical University (2023–021) and the Chinese Clinical Trial Registry in 2023 (ChiCTR2300068834). The studies were conducted in accordance with the local legislation and institutional requirements. The participants provided their written informed consent to participate in this study.

## Author contributions

AC, FL, and SM conceived the presented idea, developed the framework, and wrote the manuscript. AC, LR, and KW were involved in the data collection. YT and PL provided critical feedback and contributed to the final version. All authors contributed to the article and approved the submitted version.

## Funding

This research was funded by Chongqing Social Science Planning Project (2021PY27); Chongqing Graduate Joint Training Base (lpjd202204); Chongqing Medical University Future Medical Youth Innovation Team Development Support Plan (W0006).

## Conflict of interest

All the authors declare that the research was conducted in the absence of any commercial or financial relationships that could be construed as a potential conflict of interest.

## Publisher’s note

All claims expressed in this article are solely those of the authors and do not necessarily represent those of their affiliated organizations, or those of the publisher, the editors and the reviewers. Any product that may be evaluated in this article, or claim that may be made by its manufacturer, is not guaranteed or endorsed by the publisher.
