# Supplementary material for: Analysis of the relationship between body habitus and frailty of community adults in Chongqing: a cross-sectional survey study
Source: Front Public Health. 2023 Sep 8;11:1189173. doi: 10.3389/fpubh.2023.1189173 (PMC10516556; doi:10.3389/fpubh.2023.1189173)
Supplement: Supplementary file 1 [file Table_6.DOCX]

**Appendix 1**

| **Sociodemographic factors** | | | | | |
| --- | --- | --- | --- | --- | --- |
| 1. Your gender | Male Female | | | | |
| 1. Your age | Years | | | | |
| 1. Profession | Self-employed Company Enterprise Health Care Student Retiree | | | | |
| 1. Education background | Primary school and below Middle school High school  Undergraduate Postgraduate and above | | | | |
| 1. Monthly incomes (RMB,Yuan) | ≤1000 1001-3449 3500-9999 ≥10000 | | | | |
| 1. Marital status | Single Married Divorced Widowed | | | | |
| **Body habitus** | | | | | |
| 1. Height | Measurements were taken using a tape measure with the participant standing relaxed with the back of the head, shoulder blades, hips, and heels in contact with the vertical board, accurate to 1 cm in height | | | | |
| 1. Weight | Measure body weight to the nearest 1000 grams on a calibrated scale | | | | |
| 1. Waist circumference | The participants stand in a vertical position, with their feet separated by about 30cm naturally, so that the weight is evenly distributed, breathing smoothly, and avoiding inhalation; use a soft ruler to surround the length of the thinnest part of the waist in the horizontal direction. | | | | |
| 1. Hip circumference | Participants took a vertical standing position with their feet naturally separated by about 30 cm to distribute their weight evenly, breathe smoothly and avoid aspiration; a soft ruler was used for the length of a circle along the horizontal direction around the widest part of the hips. | | | | |
| 1. Body mass index (BMI) | BMI = weight/height^2^, the result is retained to two decimal places | | | | |
| 1. Waist-hip ratio (WHR) | WHR=(WC/HC)*100%, the result is retained to two decimal places | | | | |
| 1. Body fat percentage | R=1.2*BMI+0.23*Age -5.4-10.8*sex (1 for men, 0 for women), the result is retained to two decimal places | | | | |
| 1. Body roundness index (BRI) | BRI= WC/BMI, the result is retained to two decimal places | | | | |
| 1. A body shape index (ABSI) | ASBI=(WC)/((BMI)2/3×height1/2, the result is retained to two decimal places | | | | |
| **Lifestyle** |  | | | | |
| 1. Smoking(1) | Yes (continuous or cumulative smoking for 6 months or more)  No | | | | |
| 1. Drinking alcohol(2) | Moderate drinking(≤1 glass/day for women, ≤2 glasses/day for men)  Heavy drinking (women >1 glass/day, men >2 glasses/day)  No | | | | |
| 1. Daily exercise(3) | physical activity was defined as ≥150 minutes/week of moderate-intensity or ≥75 minutes/week of high-intensity physical activity | | | | |
| 1. Comorbidities | Yes (defined as self-reported diagnosed chronic diseases such as hypertension, diabetes, coronary heart disease, cerebrovascular disease, chronic obstructive pulmonary disease, joint diseases, etc.)  No | | | | |
| 1. Active weight loss behavior in the past year | Yes No | | | | |
| **Short Nutritional Assessment Questionnaire, SNAQ(4)** | | | | | |
|  | A=1 | B=2 | C=3 | D=4 | F=5 |
| 1. My appetite | Very poor | Poor | Fair | Good | Very good |
| 1. Meal and satiety status | feel full after eating a small amount | feel full after eating 1/3, | feel full after eating 1/2 | feel full after eating completely | feel full less often |
| 1. Food taste | Very poor | Poor | Fair | Good | Very good |
| 1. the Average number of meals per day | <1 meal | 1 meal | 2 meals | 3 meals | >3 meals |
| 1. Total scores |  | | | | |
| **Dietary Habits Assessment Scale(5)** | | | | | |
| 1. meal timing | Irregular=1 regular=2 | | | | |
| 1. breakfast habits | Never=1 Occasionally=2 Daily=3 | | | | |
|  | Eating Frequency of the following foods per week | | | | |
|  | None | 1~2 days | 3~4 days | 5~6 days | Everyday |
| 1. Eating frequency of staple food | 1 | 2 | 3 | 4 | 5 |
| 1. Eating frequency of whole grains | 1 | 2 | 3 | 4 | 5 |
| 1. Eating frequency of vegetables | 1 | 2 | 3 | 4 | 5 |
| 1. Eating frequency of fruit | 1 | 2 | 3 | 4 | 5 |
| 1. Eating frequency of poultry | 1 | 2 | 3 | 4 | 5 |
| 1. Eating frequency of meat | 1 | 2 | 3 | 4 | 5 |
| 1. Eating frequency of fish and its products | 1 | 2 | 3 | 4 | 5 |
| 1. Eating frequency of eggs and its products | 1 | 2 | 3 | 4 | 5 |
| 1. Eating frequency of beans and its products | 1 | 2 | 3 | 4 | 5 |
| 1. Eating frequency of milk and its products | 1 | 2 | 3 | 4 | 5 |
| 1. Total Scores |  | | | | |
| **Fried’s Frailty Scale(6)** | | | | | |
| 1. Exhaustion | Self-reported: at least 3 days/week | | | | |
| 1. Physical activity | Assessed using the Physical Activity Scale for the Elderly (PASE); or Kcals/week: <383 Kcals/week for males, <270 Kcals/week for females | | | | |
| 1. Walk time | >7 seconds to travel 4.57m (15 feet) on a known route | | | | |
| 1. Grip strength | Hand Grip: <5.85 Kg (12.89 lbs) for males; <3.37Kg (7.43 lbs) for females | | | | |
| 1. Weight loss | Baseline: >4.5kg (10 lbs) lost unintentionally in prior year | | | | |
| If a participant is unable to walk due to a fracture or injury, their pre-fracture/injury activity level should be reported. Meeting 1-2 of the criteria indicates pre-frailty while meeting 3 or more criteria indicates frailty. | | | | | |

**References**

1. NHIS - National Health Interview Survey [Internet]. 2023. Available from: https://www.cdc.gov/nchs/nhis/index.htm

2. World Health Organization. Global strategy to reduce the harmful use of alcohol. 2010;38.

3. World Health Organization. Global recommendations on physical activity for healt. 2010;56.

4. Kruizenga HM, Seidell JC, de Vet HCW, Wierdsma NJ, van Bokhorst–de van der Schueren MAE. Development and validation of a hospital screening tool for malnutrition: the short nutritional assessment questionnaire (SNAQ©). Clinical Nutrition. 2005 Feb 1;24(1):75–82.

5. Dietary Guidelines for Chinese Residents(2016) [Internet]. Available from: http://dg.cnsoc.org/article/2016b.html

6. Fried LP, Tangen CM, Walston J, Newman AB, Hirsch C, Gottdiener J, et al. Frailty in Older Adults: Evidence for a Phenotype. The Journals of Gerontology: Series A. 2001 Mar 1;56(3):M146–57.
